# High-Efficiency Lead-Free BNT-Based Relaxor Ferroelectrics via Synergistic A/B-Site Substitution for Enhanced Energy Storage and Stability

**DOI:** 10.3390/ma18235259

**Published:** 2025-11-21

**Authors:** Wenjie Zhou, Tao Du, Changbai Long

**Affiliations:** 1 School of Advanced Materials and Nanotechnology, Xidian University, Xi’an 710126, China; 23141110645@stu.xidian.edu.cn; 2 State Key Laboratory for Mechanical Behavior of Materials, Xi’an Jiaotong University, Xi’an 710049, China

**Keywords:** Bi_0.5_Na_0.5_TiO_3_, lead-free ceramics, energy storage performance, dielectric capacitors

## Abstract

High-efficiency, lead-free dielectrics are sought for pulsed-power capacitors, yet pristine Bi_0.5_Na_0.5_TiO_3_ (BNT) suffers from large remanence, high coercivity, and limited breakdown strength. Here, we report (1 − x)Bi_0.5_Na_0.5_Ti_0.97_Nb_0.03_O_3_-xSr_0.85_Ba_0.15_Ta_0.5+0.02x_Al_0.5−0.02x_O_3_ (BNTNb–SBTA, x = 0–0.15) ceramics synthesized via solid-state reaction, achieving enhanced relaxor ferroelectric behavior through multi-cation substitution at A- and B-sites. X-ray diffraction confirms a pure perovskite solid solution, while scanning electron microscopy reveals grain refinement, suppressing oxygen vacancies and boosting the breakdown strength. Raman and dielectric analyses evidence strengthened relaxor behavior, accompanied by loop slimming and a systematic rise in breakdown strength. The composition x = 0.10 achieves the best trade-off, delivering *W_rec_* = 3.357 J cm^−3^ and *η* = 90.5% at *E_b_* = 240 kV cm^−1^. Robust operational stability is demonstrated with small variations of *W_rec_*/*η* over 0.1–200 Hz, 25–175 °C, and 10^6^ cycles. Pulsed tests show fast discharge (∼26 ns) with *W_d_* = 0.826 J cm^−3^ at ∼90% efficiency under moderate fields. These results indicate that synergistic A/B-site disorder (Sr/Ba on A-site; Ta/Al with Nb on B-site), combined with microstructural densification, effectively minimizes *P_r_* while elevating *E_b_*, enabling high-efficiency energy storage under practical operating conditions.

## 1. Introduction

In recent years, escalating global energy consumption and environmental concerns have intensified the demand for sustainable and efficient energy storage technologies [1]. Dielectric capacitors stand out among various energy storage devices, such as batteries and electrochemical capacitors, due to their ultrahigh charge–discharge rates (on the order of nanoseconds), exceptional power density (up to 10^8^ W kg^−1^), superior fatigue endurance (>10^6^ cycles), and excellent thermal stability, making them indispensable for pulsed power systems in applications like electric vehicles, aerospace, medical devices, and military equipment [2,3]. However, traditional lead-based ceramics, such as Pb(Zr,Ti)O_3_ (PZT) and its derivatives, dominate the market owing to their superior energy storage properties, but their toxicity poses significant environmental and health risks, prompting urgent calls for lead-free alternatives [4,5,6].

Among lead-free candidates, Bi_0.5_Na_0.5_TiO_3_ (BNT)-based ceramics have emerged as particularly promising due to their high intrinsic saturation polarization (P_s_ ~ 40–43 μC cm^−2^) stemming from the Bi^3+^ lone electron pair, coupled with a high Curie temperature (T_c_ ~ 320 °C), offering environmental compatibility and suitability for high-temperature operations [7,8]. However, pure BNT exhibits limitations including high remnant polarization (*P_r_* ~ 38 μC cm^−2^) [9], large coercive field (*E_c_* ~ 70–73 kV cm^−1^) [10], and defects from Bi/Na volatility during sintering, which compromise breakdown strength (*E_b_* ~ 60–100 kV cm^−1^) and energy storage efficiency (*η* ~ 50%) [11]. To address these challenges, extensive strategies—such as A or/and B-site doping [12,13,14,15,16,17], binary/ternary solid solutions [18,19,20,21,22], and microstructural engineering [23,24,25]—have been employed to induce relaxor ferroelectric (RFE) or relaxor antiferroelectric (RAFE) behavior [26], characterized by slim polarization-electric field (P-E) hysteresis loops, reduced P_r_, and enhanced *E_b_*, thereby achieving recoverable energy densities (*W_rec_*) up to 7–12 J cm^−3^ in bulk ceramics with *η* > 85–95% [27]. Additionally, multilayer ceramic capacitor (MLCC) architectures offer enhanced breakdown strength and energy density through interface engineering and optimized layer design [28]. Despite progress in BNT-based energy storage ceramics, existing approaches face key limitations. Systems relying solely on A-site modifications often achieve loop slimming but struggle with leakage and limited breakdown strength at moderate fields. Conversely, single B-site substitution strategies (using Ta or Al independently) either excel at high-field robustness but sacrifice polarization difference, or enhance loop characteristics but introduce charge compensation challenges. Furthermore, most high-performance systems reported to date require impractically high operating fields (600–800 kV cm^−1^), limiting their applicability in real-world pulsed power devices.

A/B-site co-modification represents a synergistic strategy in BNT ceramics [29,30,31], coupling A-site disorder (e.g., Ba^2+^/Sr^2+^/K^2+^/La^3+^ replacing Bi^3+^/Na^+^) with B-site octahedral tuning to push the lattice toward an ergodic/non-ergodic relaxor or RAFE state, maximizing Δ*P* = *P*_max_ − *P_r_* while minimizing losses and boosting *E_b_* through refined microstructure and defect reduction [32,33]. On the A-site, tolerance-factor adjustment and random fields suppress long-range R3c order and lower P_r_/E_c_ [34]; the B-site then sets the loop shape and leakage via substitutions such as Sn^4+^, Zr^4+^, Nb^5+^, Ta^5+^, Mg^2+^, Cu^2+^, Ni^2+^, or complex ions like [Nb_0.5_Al_0.5_]^4+^ [35,36]. In particular, Al^3+^ substitution (direct Al→Ti or via BiAlO_3_/BaAl_0.5_Nb_0.5_O_3_) introduces strong compositional disorder that slims P-E loops and promotes relaxor/AFE-like behavior [37,38]. Because Al^3+^ is acceptor-type, it should be paired with donor co-dopants (e.g., Nb^5+^ [38,39,40]) or oxygen-vacancy control to avoid leakage [41]. By contrast, Ta^5+^ (donor) on the Ti site and Ta-bearing complex perovskites reduces oxygen vacancies (V_O_) [42], raises electrical homogeneity and *E_b_* [43], and stabilizes double/relaxor loops with lower P_r_, which often translates to higher *W_rec_* and efficiency at large fields, exemplified by (1 − x)[0.90NaNbO_3_ − 0.10Bi(Mg_0.5_Ta_0.5_)O_3_] − x(Bi_0.5_Na_0.5_)_0.7_Sr_0.3_TiO_3_ (x = 0.25) yielding *W_rec_* = 8 J cm^−3^ and *η* = 90.4% at *E_b_* = 800 kV cm^−1^, highlighting thermal stability (20 to 200 °C) and fast discharge (∼32 ns) [44]. It is speculated that, A-site (Sr/Ba) + B-site (Al + Nb or Ta) pairs are high-yield recipes. Al-centered routes excel at loop slimming and large ΔP, while Ta-centered routes excel at leakage suppression and high-field robustness—both benefiting further from microstructural densification, sub-micron grains, and core–shell/blocking-layer architectures, positioning co-modified BNT as promising for lead-free pulsed power applications in electric vehicles, aerospace, and renewable energy storage.

**Scheme 1:**
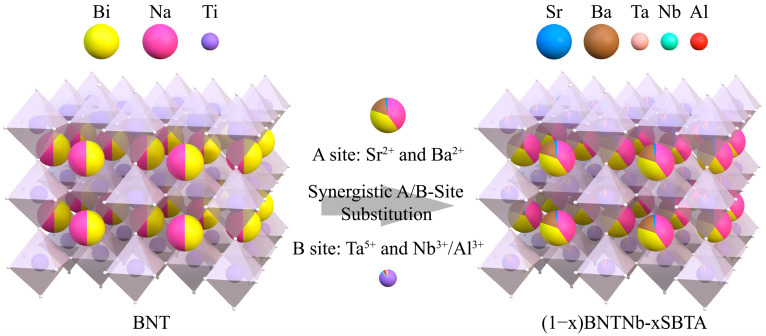
Schematic representation of the crystal structures illustrating synergistic A-site and B-site substitution in the (1 − x)BNTNb-xSBTA solid solution system.

In this work, we address these challenges through a novel quaternary lead-free relaxor ferroelectric system as shown in Scheme 1: (1 − x)Bi_0.5_Na_0.5_Ti_0.97_Nb_0.03_O_3_-xSr_0.85_Ba_0.15_Ta_0.5+0.02x_Al_0.5−0.02x_O_3_ (abbreviated as (1 − x)BNTNb-xSBTA, x = 0, 0.05, 0.1, 0.15, 0.2). In this notation, BNTNb denotes Nb-modified Bi_0.5_Na_0.5_TiO_3_ (Bi_0.5_Na_0.5_Ti_0.97_Nb_0.03_O_3_), and SBTA denotes the complex perovskite Sr_0.85_Ba_0.15_Ta_0.5+0.02x_Al_0.5−0.02x_O_3_, which introduces Sr^2+^/Ba^2+^ on the A-site and Ta^5+^/Al^3+^ on the B-site. The novelty of this composition lies in three synergistic design strategies. First, we employ Nb-modified BNT as the base matrix to establish initial relaxor characteristics and donor-type doping for oxygen vacancy suppression. Second, we introduce a complex perovskite end-member (SBTA) featuring dual A-site cations (Sr^2+^/Ba^2+^) that adjust the tolerance factor and create random local fields to promote ergodic relaxor behavior, combined with a variable-stoichiometry B-site pair (Ta^5+^/Al^3+^) that simultaneously induces strong compositional disorder for loop slimming while maintaining donor-acceptor charge balance to prevent leakage. Third, this multi-cation substitution strategy naturally drives grain refinement through solute drag mechanisms, increasing grain boundary density to enhance breakdown strength without requiring additional processing steps. This integrated approach combines the complementary advantages of Al-centered routes (excellent loop slimming and large polarization difference) with Ta-centered strategies (superior leakage suppression and high-field robustness) in a single system, which has not been systematically explored in BNT-based ceramics. The Nb concentration of 3 mol% in the BNT matrix was selected to provide optimal donor doping within established solubility limits [45], reducing oxygen vacancy concentration while enhancing breakdown strength. The SBTA end-member employs an Sr:Ba ratio of 0.85:0.15 to balance relaxor behavior and phase stability, combined with a coupled Ta:Al variation (Ta_0.5+0.02x_Al_0.5−0.02x_) that synergistically integrates donor doping effects with relaxor-promoting disorder [46]. Through comprehensive structural, dielectric, and ferroelectric characterization, we demonstrate that the optimized composition achieves high energy storage density (*W_rec_* = 3.357 J cm^−3^) with exceptional efficiency (*η* = 90.5%) at moderate and practically accessible fields (240 kV cm^−1^), alongside robust thermal stability (25–175 °C), frequency independence (0.1–200 Hz), fatigue resistance (10^6^ cycles), and ultrafast discharge capability (approximately 26 ns). These results establish a viable pathway for high-performance lead-free dielectric capacitors in practical pulsed power applications.

## 2. Materials and Methods

### 2.1. Sample Preparation

(1 − x)Bi_0.5_Na_0.5_Ti_0.97_Nb_0.03_O_3_-xSr_0.85_Ba_0.15_Ta_0.5+0.02x_Al_0.5−0.02x_O_3_ (x = 0, 0.05, 0.1, 0.15, 0.2) ceramics were synthesized using the conventional solid-state reaction method. High-purity starting materials, including Bi_2_O_3_ (99%), Na_2_CO_3_ (99.8%), TiO_2_ (99%), Nb_2_O_5_ (99.5%), SrCO_3_ (99%), BaCO_3_ (99.8%), Ta_2_O_5_ (99.5%), and Al_2_O_3_ (99%) (Aladdin Biochemistry and Technology Co., Ltd., Shanghai, China), were dried at 80 °C for 12 h prior to use. The reagents were weighed in stoichiometric proportions, mixed thoroughly, and subjected to primary ball milling (Nanjing Nanda Instrument Co., Ltd., Nanjing, China) in nylon jars at 400 rpm for 12 h, followed by drying at 80 °C for 4 h. The resulting powders were then calcined in a muffle furnace (KSL-1400X, Hefei Kejing, Hefei, China) at 800 °C for 4 h. Subsequently, the calcined powders underwent secondary ball milling at 400 rpm for 12 h and were dried again at 80 °C for 4 h. For pellet formation, 0.2 g of dried powder was weighed and uniaxially pressed manually in a 10 mm diameter die to obtain green compacts. These compacts were encased in rubber sleeves, evacuated, and densified via cold isostatic pressing at 250 MPa for 10 min. Finally, the green bodies corresponding to x = 0, 0.05, 0.1, and 0.15 were sintered at 1070 °C for 3 h in air. This sintering temperature was selected based on optimization trials and guidance for BNT-based ceramics with A-site substitutions, which demonstrated that temperatures in the 1050–1100 °C range effectively balance densification with minimized Bi/Na volatilization and controlled grain growth [47,48]. For x = 0.2, the samples exhibited poor densification and were excluded from subsequent characterizations. The sintered pellets were polished to a thickness of 0.1–0.2 mm, and silver electrodes were applied on both polished surfaces for electrical measurements.

### 2.2. Characterization Methods

The crystal structures of the ceramic samples were characterized using an X-ray diffractometer (XRD; D8 Advance, Bruker, Berlin, Germany). The microstructures were investigated via field-emission scanning electron microscopy (FE-SEM; Apero HiVac, Thermo Fisher Scientific, Waltham, MA, USA). The bulk densities of the sintered ceramics were measured using the Archimedes method in distilled water at room temperature. Laser confocal micro-Raman spectroscopy (Renishaw inVia, Wotton-under-Edge, UK) was employed to gain deeper insights into the vibrational modes and local structural evolution of the materials. Ferroelectric properties, including P-E hysteresis loops measured at 1 Hz, as well as temperature, frequency, and fatigue stability, were evaluated using a ferroelectric analyzer (TF Analyzer 3000, aixACCT, Aachen, Germany). Dielectric properties, including the frequency- and temperature-dependent dielectric constants, were determined with a dielectric temperature spectrometer (CH400-100-P8, Beijing Sinocera Functional Material Co., Ltd., Beijing, China). Charge–discharge performance was analyzed using a dielectric charge–discharge system (CFD-300, Beijing Sinocera Functional Material Co., Ltd., Beijing, China).

### 2.3. Energy Storage Parameter Calculations

#### 2.3.1. Total Energy Storage Density (*W_tot_*)

The total electrostatic energy density stored during charging is calculated as

(1) $$W_{tot}=\int_{0}^{P_{\max}}EdP$$

where *E*(*P*) is the electric field as a function of polarization during the charging branch of the unipolar loop, and *P*_max_ is the maximum polarization reached under the applied field.

#### 2.3.2. Recoverable Energy Storage Density (*W_rec_*)

The recoverable (dischargeable) portion of the stored energy is obtained from the unloading branch of the same unipolar loop and is given by

(2) $$W_{rec}=\int_{P_{r}}^{P_{\max}}EdP$$

where *P_r_* is the remanent polarization after the external field is removed. Physically, *W_rec_* corresponds to the area under the discharge curve between *P*_max_ and *P_r_*.

#### 2.3.3. Energy Storage Efficiency (*η*)

The energy storage efficiency, which reflects dielectric loss and hysteresis loss, is defined as

(3) $$\eta =\frac{W_{rec}}{W_{tot}}\times 100\%=\frac{W_{rec}}{W_{rec}+W_{loss}}\times 100\%$$

#### 2.3.4. Breakdown Electric Field (*E_b_*)

For each composition, the breakdown electric field $E_{b}$ was defined as the highest field that could be applied without electrical failure (catastrophic increase in leakage current or dielectric puncture).

#### 2.3.5. Pulsed Charge–Discharge Analysis

For the pulsed discharge tests, the discharged energy density *W_d_* and the power-related quantities were extracted from the transient voltage–current waveforms. The discharged energy density is obtained from

(4) $$W_{d}=\frac{1}{V_{samp}}\int_{t_{0}}^{t_{f}}V(t)I(t)dt$$

where *V*(*t*) and *I*(*t*) are the instantaneous voltage and current during discharge, *V_samp_* is the sample volume, and *t*_0_ − *t_f_* spans the main discharge pulse. The peak power density (PD) is estimated from

(5) $$\mathrm{PD}=\frac{V_{peak}I_{peak}}{V_{samp}}$$

The reported charge density (CD) corresponds to the maximum current per electrode area, and the quoted discharge time corresponds to the interval required to deliver the majority of *W_d_*.

## 3. Results

**Figure 1:**
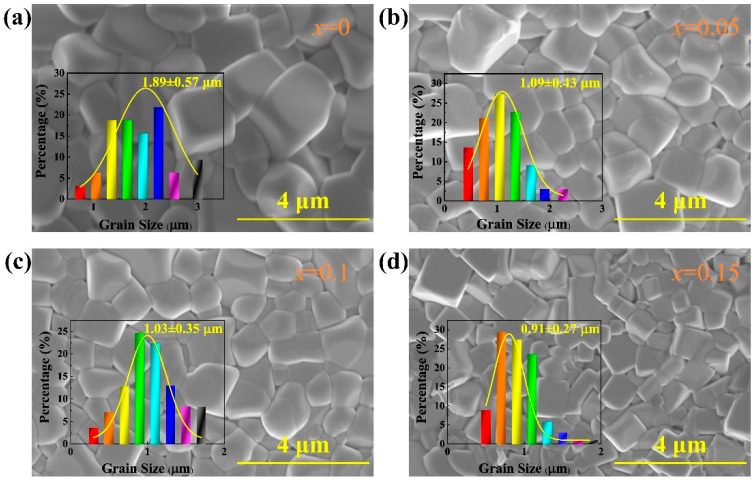
SEM images of the (1 − x)BNTNb-xSBTA ceramics with (**a**) x = 0, (**b**) x = 0.05, (**c**) x = 0.1, (**d**) x = 0.15, insets show the average grain size and the grain size distribution.

Figure 1 shows the scanning electron microscopy (SEM) images of (1 − x)BNTNb- xSBTA ceramics sintered at 1070 °C for x = 0, 0.05, 0.10, and 0.15, respectively. All samples exhibit dense microstructures with well-defined grain boundaries, minimal porosity, and no secondary phases. Statistical analysis (inset bar charts) reveals that average grain size decreases systematically with increasing SBTA content: 1.89 μm (x = 0), 1.09 μm (x = 0.05), 1.03 μm (x = 0.10), and 0.91 μm (x = 0.15). This grain refinement provides indirect evidence for oxygen vacancy suppression [49] and enhances breakdown strength by increasing grain boundary density, which acts as a scattering center for charge carriers and improves insulation resistance [50].

To quantitatively assess the densification, bulk density measurements were conducted using the Archimedes method. Table 1 summarizes the bulk density, average grain size, and breakdown strength for all compositions. The bulk density increases systematically from 5.68 g cm^−3^ (x = 0) to 5.86 g cm^−3^ (x = 0.15), demonstrating progressive densification with increasing SBTA content. This trend correlates directly with the observed grain refinement and enhancement in breakdown strength. The systematic increase in bulk density with SBTA doping demonstrates that the addition of SBTA not only refines the grain size but also promotes better densification of the ceramic matrix. With increasing SBTA doping content, the synergistic combination of enhanced bulk density and reduced grain size creates a microstructure with higher grain boundary density. These grain boundaries serve as effective scattering centers for charge carriers, thereby suppressing oxygen vacancy formation, reducing leakage current pathways, and increasing the breakdown strength. This microstructural optimization through SBTA incorporation improves the overall insulation resistance and energy storage performance of the ceramics, consistent with mechanisms observed in other BNT-based systems with refined and densified microstructures [51].

**Figure 2:**
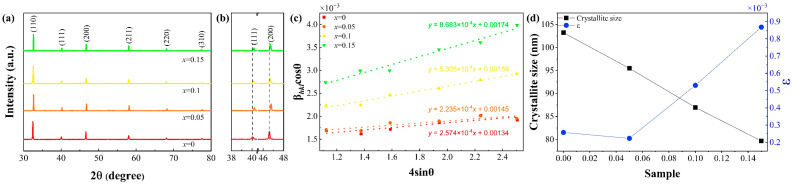
(**a**) XRD patterns of (1 − x)BNTNb-xSBTA ceramics with different compositions, (**b**) Magnified views of the (111) and (200) characteristic peaks, (**c**) Williamson-Hall plots of βcosθ versus 4sinθ for (1 − x)BNTNb-xSBTA ceramics with varying SBTA content (x = 0, 0.05, 0.1, 0.15), (**d**) Compositional dependence of crystallite size (black squares, left axis) and lattice microstrain (blue circles, right axis) extracted from Williamson-Hall analysis for (1 − x)BNTNb-xSBTA ceramics.

From the X-ray diffraction (XRD) patterns (Figure 2a) of (1 − x)BNTNb-xSBTA ceramics recorded at room temperature in the 2θ range of 30–80°, all diffraction peaks can be indexed to a pure perovskite structure, with no detectable secondary phases across the investigated compositions (x = 0–0.15). This confirms the complete solid solubility of the SBTA component within the BNTNb lattice, forming a homogeneous solid solution without phase segregation, consistent with the formation of perovskite-based relaxor ferroelectrics in BNT systems [52]. A magnified view of the (111) and (200) diffraction peaks in the 2θ range of 38–48° is presented in Figure 2b. With increasing SBTA doping content, the evolution of these characteristic peaks reflects competing effects from simultaneous A-site and B-site substitutions. At the A-site, Bi^3+^ (1.03 Å, CN = 8) and Na^+^ (1.18 Å, CN = 8) are partially replaced by Sr^2+^ (1.26 Å, CN = 8) and Ba^2+^ (1.42 Å, CN = 8), both possessing larger ionic radii, which tends to expand the lattice and shift diffraction peaks toward lower 2θ angles. Conversely, at the B-site, the average ionic radius of [Ti_0.97_Nb_0.03_]^4+^ (approximately 0.606 Å) in the BNTNb matrix is replaced by the smaller [Ta_0.5+0.02x_Al_0.5−0.02x_]^4+^ (approximately 0.588 Å), driven by the incorporation of Ta^5+^ (0.64 Å, CN = 6) and Al^3+^ (0.535 Å, CN = 6), which contracts the lattice and shifts peaks toward higher 2θ angles. The non-monotonic lattice parameter evolution observed in the diffraction patterns results from the delicate balance between these two counteracting mechanisms, with the B-site contraction effect dominating at low SBTA concentrations and the A-site expansion effect becoming more pronounced at higher concentrations. Such lattice shrinkage disrupts long-range ferroelectric order, promoting the formation of polar nanoregions (PNRs) and potentially enhancing the *E_b_* by improving insulation and reducing domain wall mobility [53,54,55,56,57,58]. Williamson-Hall analysis was conducted to quantitatively evaluate microstructural changes using the equation βcosθ = kλ/D + 4εsinθ. where β represents the corrected full width at half maximum, θ is the Bragg angle (in radians), k is the Scherrer constant (0.9), λ is the X-ray wavelength (1.5406 Å), D is the volume-weighted crystallite size, and ε denotes the lattice microstrain. As illustrated in Figure 2c,d, crystallite size decreases systematically from 103 nm at x = 0 to 80 nm at x = 0.15, while lattice microstrain increases from 0.2574 × 10^−3^ to 0.8683 × 10^−3^. The grain refinement results from solute drag and Zener pinning effects induced by SBTA incorporation, which inhibit grain growth during sintering. The substantial microstrain enhancement originates from ionic radius mismatch at both A-sites (Sr^2+^/Ba^2+^ versus Bi^3+^/Na^+^) and B-sites (Ta^5+^/Al^3+^ versus Ti^4+^/Nb^5+^), along with coherency strain at phase interfaces. These microstructural modifications contribute synergistically to enhanced breakdown strength and relaxor ferroelectric characteristics favorable for energy storage performance. Furthermore, energy-dispersive X-ray spectroscopy (EDS) mapping (Figure S1) reveals a homogeneous distribution of all constituent elements in the x = 0.1 ceramic samples. This uniformity indicates that Sr^2+^, Ba^2+^, Ta^5+^, Al^3+^ and Nb^5+^ ions have been successfully incorporated into the crystal lattice, substituting for host cations at the A- and B-sites, which is consistent with the phase purity and structural integrity confirmed by XRD analysis.

**Figure 3:**
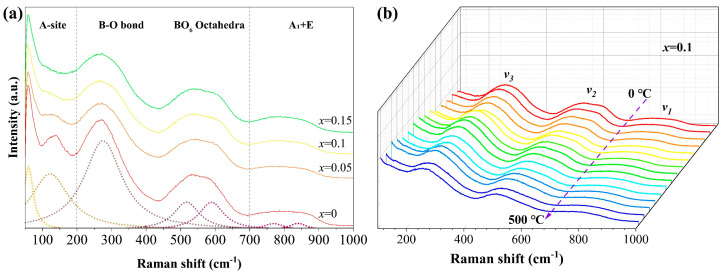
(**a**) Raman spectra of (1 − x)BNTNb-xSBTA ceramics with different compositions, (**b**) Temperature-dependent evolution of Raman peaks for the x = 0.1 composition in the range of 0 to 500 °C.

To gain a deeper understanding of the vibrational modes and local structural evolution of (1 − x)BNTNb-xSBTA ceramics, Raman spectra of ceramics with different components were acquired at room temperature over the wavenumber range of 50–1000 cm^−1^. The low-wavenumber region (<200 cm^−1^) corresponds to vibrational modes involving A-site cations (Bi^3+^, Na^+^, Sr^2+^, and Ba^2+^), while the intermediate range (200–450 cm^−1^) is dominated by B–O bending modes. The high-wavenumber region (450–700 cm^−1^) arises from stretching vibrations of the BO_6_ octahedra, and peaks above 700 cm^−1^ reflect overlapping A_1_ + E longitudinal optical (LO) modes [59]. With increasing SBTA doping content (x = 0–0.15), the peak intensity near 135 cm^−1^ gradually disappears, attributable to restricted displacements of Bi^3+^ and Na^+^ ions at the A-site due to the incorporation of larger Sr^2+^ and Ba^2+^ cations [60]. Similarly, the peak at ~270 cm^−1^, associated with B-O bending, exhibits smoothing, indicative of enhanced cation disorder where Bi^3+^, Na^+^, Sr^2+^, and Ba^2+^ randomly occupy A-sites, while Ti^4+^, Nb^5+^, Ta^5+^, and Al^3+^ substitute at B-sites [61]. The broadening and flattening of the ~570 cm^−1^ peak, linked to BO_6_ octahedral stretching, further signify local distortions induced by SBTA doping. These spectral evolutions underscore the development of dielectric relaxation, characterized by disordered local structures and PNRs, which influence the ferroelectric and dielectric properties of the ceramics. Figure 3b illustrates the temperature-dependent Raman spectra for the x = 0.1 composition (0.9BNTN–0.1SBTA) from 0 to 500 °C, revealing minimal shifts or intensity changes across the measured range. The temperature insensitivity of the spectra indicates the absence of macroscopic phase transitions, a hallmark of relaxor ferroelectrics. This behavior is primarily governed by the persistence of dynamic PNRs,—thermally fluctuating nanoscale polar clusters that reorient reversibly without coalescing into macroscopic domains even at elevated temperatures beyond the dielectric maximum temperature (*T_m_*). Such nanoscale heterogeneity contributes to the observed enhanced dielectric stability in the x = 0.1 composition, suppressing long-range ferroelectric ordering and promoting slim P-E hysteresis loops essential for high energy storage efficiency.

**Figure 4:**
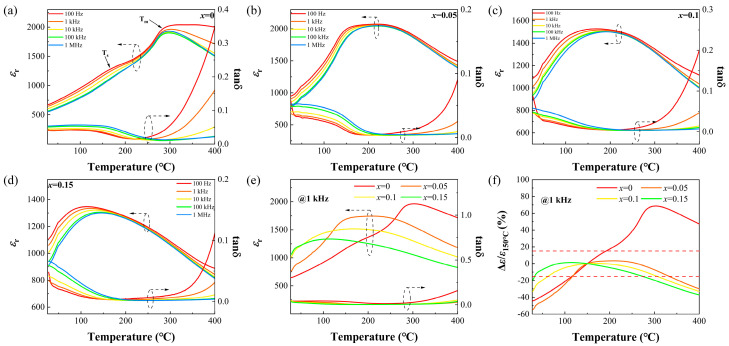
(**a**–**d**) Temperature-dependent dielectric spectra of (1 − x)BNTNb-xSBTA ceramics with different compositions, (**e**) Dielectric spectra of (1 − x)BNTNb-xSBTA ceramics with different compositions measured at 1 kHz, (**f**) Thermal stability of the dielectric constant (*ε_r_*) of (1 − x)BNTNb-xSBTA ceramics.

To investigate the dielectric behavior and its evolution with SBTA doping, the temperature-dependent dielectric permittivity (*ε_r_*) and dielectric loss tangent (tan *δ*) for (1 − x)BNTNb-xSBTA ceramics with different compositions were measured over a frequency range of 0.1 kHz to 1 MHz from 25 to 400 °C (Figure 4a–d). Each composition displays two characteristic dielectric anomalies: a shoulder peak at T_s_ (associated with the onset of thermal evolution of PNRs, where nanoscale polar clusters begin to grow and interact more strongly due to cooling-induced correlations) and a maximum peak at *T_m_* (reflecting the diffuse phase transition). These anomalies are consistent with the thermal dynamics of PNRs in rhombohedral R3c and tetragonal P4bm structures typical of BNT-based relaxor ferroelectrics [62,63]. Moreover, both T_s_ and *T_m_* exhibit pronounced frequency dispersion, with peaks shifting to higher temperatures and broadening as frequency increases, confirming the relaxor ferroelectric nature of the ceramics.

As shown in Figure 4e, increasing SBTA doping content leads to a substantial reduction in *ε_r_* and a shift in the dielectric peaks toward lower temperatures. At 1 kHz, the maximum *ε_r_* decreases from ~1962 for the undoped BNTNb (x = 0) to ~1516 for the x = 0.1 composition, reflecting suppressed long-range ferroelectric order and enhanced local disorder from multi-cation substitution at A- and B-sites. Concurrently, tan *δ* decreases monotonically with higher x, indicating reduced electrical conductivity and hysteresis losses, which favors improved energy storage efficiency (*η*). To evaluate thermal stability, the temperature coefficient of capacitance (TCC) was calculated relative to 150 °C using the formula [64,65,66]:

(6) $$TCC_{150{}^{\circ}C}=\frac{{\varepsilon^{\prime }}_{T}-{\varepsilon^{\prime }}_{150{}^{\circ}C}}{{\varepsilon^{\prime }}_{150{}^{\circ}C}}\times 100\%=\frac{\Delta \varepsilon^{\prime }}{{\varepsilon^{\prime }}_{150{}^{\circ}C}}\times 100\%$$

where ${\varepsilon^{\prime }}_{T}$ and are the dielectric constants at a certain temperature and benchmark temperature, respectively. Figure 4f demonstrates progressive improvement in *TCC* stability with increasing SBTA content. Notably, the x = 0.1 composition maintains a dielectric permittivity variation of Δ*ε_r_*/*ε*_150°C_ ≤ ±15% over the broad range of 63–307 °C, underscoring the role of SBTA-induced PNRs in promoting diffuse phase transitions and enhancing temperature-insensitive dielectric response.

**Figure 5:**
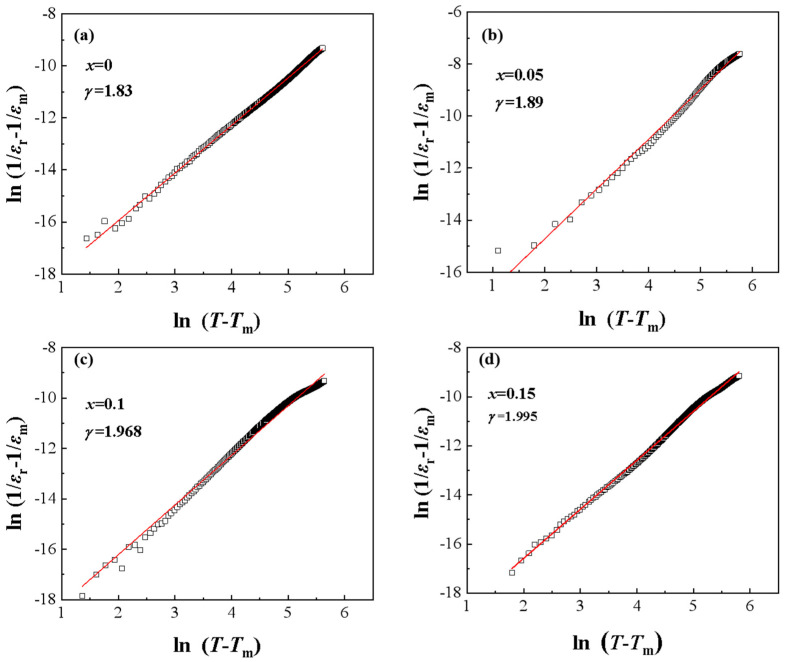
(**a**–**d**) Linear fitting curves of ln(1/*ε_r_* − 1/*ε_m_*) versus ln(*T* − *Tₘ*) for (1 − x)BNTNb-xSBTA ceramics with different compositions.

The degree of diffuseness in the phase transition of the ceramics was quantified using the modified Curie–Weiss law [67]:

(7) $$\frac{1}{\varepsilon^{\prime }}-\frac{1}{{\varepsilon^{\prime }}_{m}}=\frac{{\left(T-T_{m}\right)}^{\gamma }}{C}$$

where *ε* represents the dielectric constant at temperature *T*, *ε_m_* is the maximum permittivity at the temperature *T_m_* of the dielectric peak, *C* is the Curie constant, and *γ* is the diffuseness parameter ranging from 1 (normal ferroelectric behavior) to 2 (ideal relaxor ferroelectric with complete diffuseness). Values of *γ* closer to 2 indicate stronger relaxor characteristics, associated with the dynamic evolution of PNRs (where these polar clusters fluctuate and grow in size or correlation length as temperature decreases, suppressing long-range ferroelectric order) and suppressed long-range ferroelectric order. Figure 5a–d displays the modified Curie–Weiss plots for (1 − x)BNTNb-xSBTA ceramics (x = 0, 0.05, 0.10, 0.15), fitted to extract the *γ* values. The diffuseness coefficients increase progressively with SBTA doping, from 1.83 (x = 0) to 1.89 (x = 0.05), 1.968 (x = 0.10), and 1.995 (x = 0.15). This enhancement of *γ* reflects the increasing degree of structural disorder induced by multi-cation substitution at A- and B-sites, promoting nanoscale heterogeneity and PNR formation. Notably, the x = 0.10 composition exhibits a *γ* of 1.968, signifying pronounced relaxor behavior that contributes to slim P-E hysteresis loops, reduced remnant polarization, and improved energy storage efficiency by minimizing hysteresis losses.

**Figure 6:**
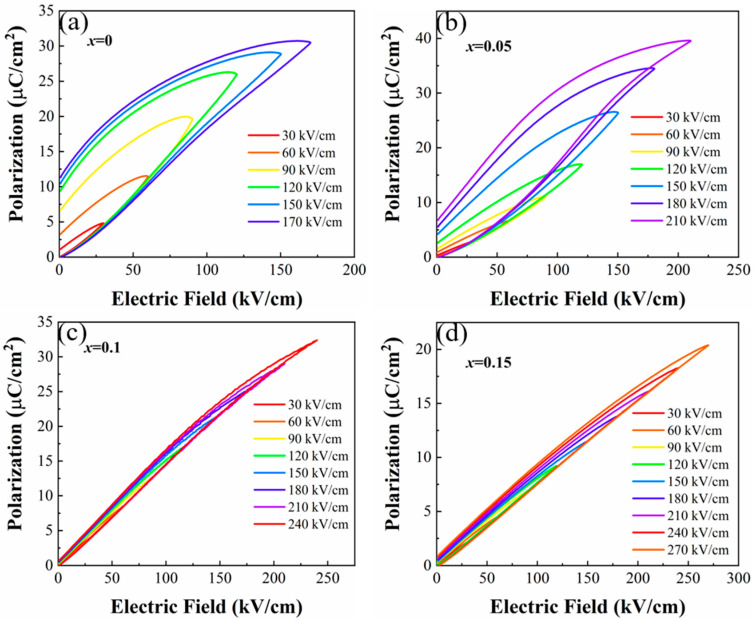
(**a**–**d**) Unipolar P-E loops of (1 − x)BNTNb-xSBTA ceramics at 1 Hz under varying electric fields.

To assess the ferroelectric response and energy storage capabilities under varying SBTA doping, unipolar P-E hysteresis loops for (1 − x)BNTNb-xSBTA ceramics were measured at room temperature and 1 Hz as a function of the applied electric field up to breakdown (Figure 6a–d). With increasing SBTA doping content, the loops progressively narrow, indicative of reduced hysteresis and enhanced relaxor ferroelectric characteristics. This slimming arises from the disruption of long-range ferroelectric domains into dynamic PNRs (nanoscale polar entities that fluctuate thermally and align reversibly under electric fields), facilitated by B-site cation migration under the electric field. The substitution of Ti^4+^ and Nb^5+^ by Ta^5+^ and Al^3+^ generates random local fields due to charge imbalances and ionic radius mismatches, further promoting nanoscale heterogeneity and PNR formation. These PNRs enable reversible transitions to long-range order under applied fields, yielding larger maximum polarization with minimal remnant polarization and energy loss. The systematic enhancement of breakdown strength with SBTA doping (Table 2) can be ascribed to two synergistic effects: suppression of oxygen vacancy formation, which mitigates defect-induced conductivity and improves insulation resistance, and progressive grain refinement, leading to a higher density of grain boundaries that scatter charge carriers and impede leakage currents. To evaluate the insulation properties and charge transport behavior of (1 − x)BNTNb-xSBTA ceramics, DC leakage current density measurements were conducted at room temperature across electric fields ranging from 0 to 100 kV/cm. As shown in Figure S1 (Supplementary Materials), the leakage current density reduces from approximately 1.7 × 10^−7^ A cm^−2^ for the undoped composition (x = 0) to approximately 5.2 × 10^−8^ A cm^−2^ for the optimized composition (x = 0.1), representing nearly an order of magnitude suppression. This progressive reduction provides direct experimental evidence for the effectiveness of our multi-cation substitution strategy in suppressing oxygen vacancy formation and improving insulation resistance.

**Figure 7:**
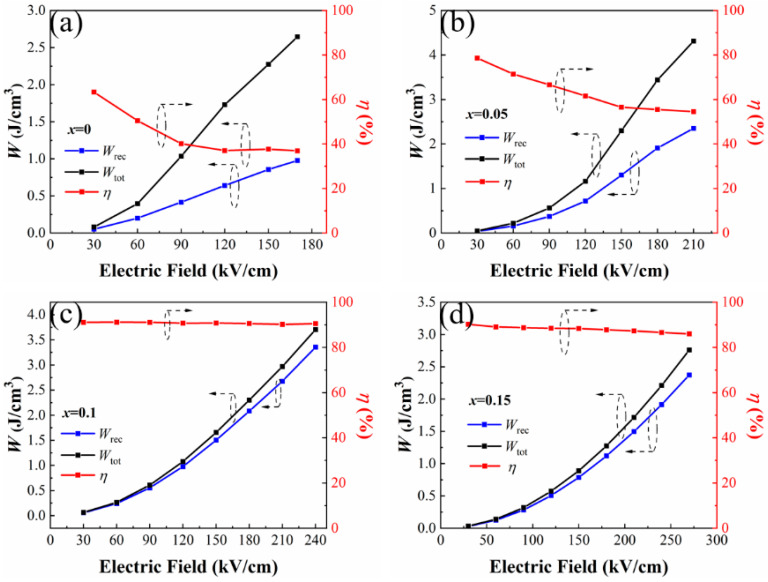
(**a**–**d**) Variations in recoverable energy density (*W_rec_*), total energy density (*W_tot_*), and energy efficiency (*η*) with electric field strength for (1 − x)BNTNb-xSBTA ceramics at room temperature.

To evaluate the energy storage performance and its evolution with SBTA doping across applied fields, Figure 7 depicts the electric field dependence of recoverable energy storage density (*W_rec_*), total energy storage density (*W_tot_*), and energy storage efficiency (*η*) for (1 − x)BNTNb-xSBTA ceramics at room temperature. With increasing SBTA doping content, both *W_rec_* and *W_tot_* exhibit monotonic enhancement across the applied field range, attributed to the progressively larger polarization difference and elevated breakdown strength enabled by refined grains and suppressed defects. Notably, *η* shows enhanced field stability in higher SBTA compositions, declining for x = 0 and 0.05 due to hysteresis from long-range order, but plateauing above 80% for x = 0.10 and 0.15 via dynamic PNRs that reduce dissipation through minimized domain motion and stronger relaxor traits. This evolution underscores the role of SBTA-induced nanoscale heterogeneity in optimizing the overall energy storage performance, facilitating slim P-E loops and low-loss behavior essential for practical dielectric capacitor applications.

**Figure 8:**
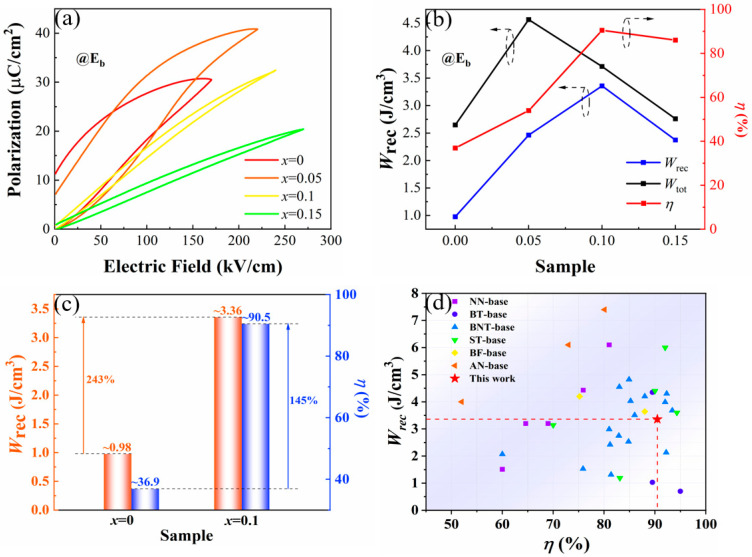
(**a**) Unipolar P-E loops of (1 − x)BNTNb-xSBTA ceramics at the breakdown electric field (*E_b_*) as a function of x content. (**b**) Composition-dependent recoverable energy density (*W_rec_*), total energy density (*W_tot_*), and energy efficiency (*η*) at *E_b_*. (**c**) Comparison of energy storage performance between x = 0 and x = 0.1 ceramics. (**d**) Performance comparison between the ceramics in this work and previously reported lead-free ceramics.

To quantify the compositional optimization of energy storage performance under maximum applied fields, Figure 8a–d summarizes the unipolar P-E hysteresis loops and key metrics for (1 − x)BNTNb-xSBTA ceramics evaluated at their respective breakdown strengths, with detailed numerical results presented in Table 2. As shown in Figure 8a, the loops narrow progressively with increasing SBTA content, reflecting diminished hysteresis due to the transition from long-range ferroelectric domains to dynamic PNRs, which suppress remnant polarization and enhance the polarization difference. The composition-dependent trends (Figure 8b and Table 2) reveal that energy storage performance initially rises with x before peaking at x = 0.10, where the balanced local disorder, grain refinement, and defect suppression minimize energy loss while maximizing recoverable energy density and efficiency. Figure 8c highlights the substantial enhancements relative to the undoped sample, with the x = 0.10 composition exhibiting a 243% increase in *W_rec_* and 145% improvement in *η*, underscoring the efficacy of SBTA doping in promoting relaxor behavior and insulation. For contextual benchmarking, Figure 8d compares the performance of this work with reported lead-free ceramics across BNT-, ST-, NN-, and AN-based systems, demonstrating that the x = 0.10 composition outperforms the majority of prior studies, particularly in achieving high efficiency under moderate fields, attributable to the synergistic nanoscale heterogeneity and microstructural tailoring that yield slim P-E loops and robust breakdown strength. Specifically, the superior performance relative to other BNT-based ceramics can be attributed to its refined grain structure and the effective suppression of deleterious defects. The pronounced grain refinement achieved in this composition yields a higher density of grain boundaries, which act as insulating barriers that scatter charge carriers and inhibit domain wall motion. This microstructural feature elevates the overall insulation resistance and helps maintain slim hysteresis loops by limiting extrinsic energy losses. Concurrently, the aliovalent co-doping with Ta^5+^ and Al^3+^ minimizes oxygen vacancy formation through internal charge compensation, thereby reducing defect-induced leakage currents. The lower concentration of oxygen vacancies bolsters the breakdown strength and ensures that high efficiency is retained under moderate electric fields. Together, these structural (grain boundary engineering) and electrical (defect mitigation) mechanisms synergistically explain why the x = 0.10 composition outperforms conventional BNT-based systems in energy storage efficiency and reliability.

**Figure 9:**
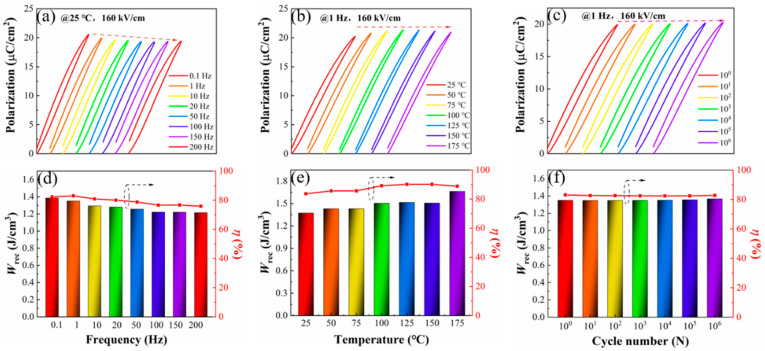
Energy storage stability of 0.9BNTNb-0.1SBTA ceramics under various conditions. (**a**–**c**) Evolution of P-E loops with (**a**) frequency, (**b**) temperature, and (**c**) cycling number, respectively. (**d**–**f**) Corresponding variations in recoverable energy density (*W_rec_*) and energy efficiency (*η*) with (**d**) frequency, (**e**) temperature, and (**f**) cycling number, respectively.

In practical applications, operational stability under varying frequency, temperature, and cycling conditions is paramount for dielectric capacitors [65]. To assess these attributes, the 0.9BNTNb-0.1SBTA ceramics were evaluated under a fixed electric field of 160 kV cm^−1^, with unipolar P-E hysteresis loops and derived energy storage metrics (*W_rec_*, *W_tot_*, and *η*) presented in Figure 9a–f. Figure 9a,d reveal the frequency dependence from 0.1 to 200 Hz at 25 °C; the loops remain slender with minimal broadening, yielding *W_rec_* = 1.30 ± 0.09 J cm^−3^ and *η* = 79 ± 3%. This frequency insensitivity stems from the relaxor nature of the material, where the dynamic PNRs (induced by SBTA multi-cation substitution) respond rapidly and reversibly to alternating fields, suppressing frequency-induced domain pinning and associated energy dissipation that would otherwise arise from sluggish domain wall motion in conventional ferroelectrics. Temperature dependence from 25 to 175 °C at 1 Hz, shown in Figure 9b,e, demonstrates robust loop integrity, with *W_rec_* = 1.37 ± 0.15 J cm^−3^ and *η* = 87 ± 3%, attributed to the thermal persistence of nanoscale heterogeneity; specifically, the PNRs maintain short-range polar order without coalescing into long-range ferroelectric domains even at elevated temperatures, thereby inhibiting sharp phase transitions and ensuring a diffuse, temperature-stable dielectric response. Fatigue resistance over 106 cycles at 1 Hz and 25 °C is depicted in Figure 9c,f, where the P-E loops exhibit negligible degradation, maintaining *W_rec_* = 1.36 ± 0.10 J cm^−3^ and *η* = 83 ± 0.01%; this endurance reflects the fatigue-resistant characteristics of the PNRs, which facilitate reversible polarization switching with minimal structural degradation or charge trapping, as opposed to the irreversible domain reorientation and defect accumulation typical in non-relaxor systems. Overall, the variations in *W_rec_* and *η* across these conditions remain within ±10–15%, highlighting the superior thermal, frequency, and cycling stability of the 0.9BNTNb-0.1SBTA composition, which arises from SBTA-induced structural disorder and enhanced insulation, positioning it as a promising candidate for reliable high-temperature capacitor applications.

**Figure 10:**
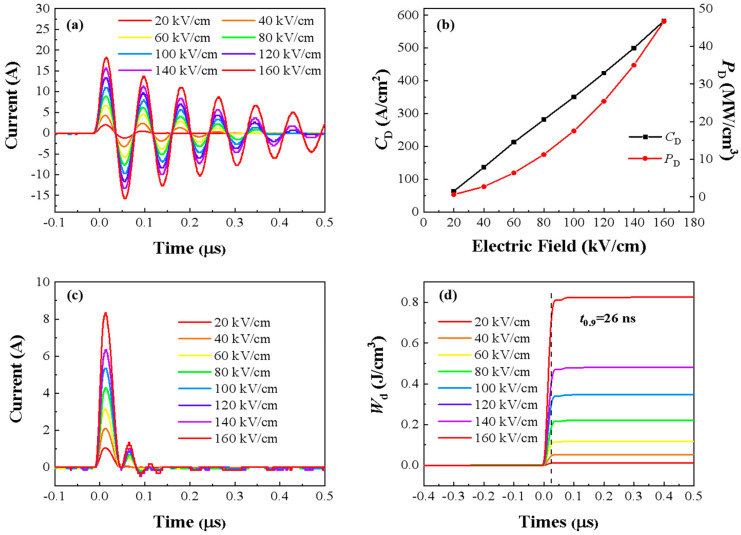
Energy storage and discharge behavior of 0.9BNTNb-0.1SBTA ceramics under electric fields ranging from 20 to 160 kV cm^−1^. (**a**) Underdamped pulsed charge–discharge current curves. (**b**) Charge density (CD) and power density (PD) values. (**c**) Overdamped pulsed charge–discharge waveforms. (**d**) Variation in discharged energy density (*W_d_*) with time.

To evaluate the practical charge–discharge capabilities of the 0.9BNTNb-0.1SBTA ceramics, pulsed discharge tests were conducted under both underdamped and overdamped conditions across electric fields from 20 to 160 kV cm^−1^, as detailed in Figure 10. In the underdamped regime (Figure 10a), the discharge waveforms maintain stable oscillatory profiles with no distortion, and the peak current (I_max_) increases from 4.23 to 18.3 A as the field rises, corresponding to a charge density (CD) enhancement from 63.7 A cm^−2^ to 582 A cm^−2^ and a power density (PD) rise from 0.6 to 46.6 MW cm^−3^ (Figure 10b). This field-dependent scaling reflects the efficient polarization reversal facilitated by dynamic PNRs, which enable rapid energy release with minimal losses in relaxor ferroelectrics. For the overdamped configuration (Figure 10c), the waveforms exhibit consistent pulse shapes, with I_max_ similarly increasing with field strength, and the discharged energy density (*W_d_*) progressing from 0.013 to 0.826 J cm^−3^ (Figure 10d). Notably, the maximum *W_d_* of 0.826 J cm^−3^ is released at 90% efficiency within ~26 ns, underscoring the ultrafast discharge kinetics driven by low hysteresis and high insulation resistance. These results demonstrate the potential of 0.9BNTNb-0.1SBTA ceramics for high-power pulsed applications, where sub-microsecond response times and power densities align with requirements for advanced electronic systems [68].

## 4. Conclusions

This work establishes a novel compositional design pathway for high-performance lead-free energy storage ceramics through synergistic A/B-site substitution in (1 − x)Bi_0.5_Na_0.5_Ti_0.97_Nb_0.03_O_3_-xSr_0.85_Ba_0.15_Ta_0.5+0.02x_Al_0.5−0.02x_O_3_. The fundamental innovation lies in the strategic integration of three complementary modification mechanisms within a single quaternary system, an approach not previously explored in BNT-based materials. Specifically, the composition combines Nb-modified BNT as a base matrix with a complex perovskite end-member featuring dual A-site cations (Sr^2+^/Ba^2+^) and a variable-stoichiometry B-site pair (Ta^5+^/Al^3+^). This design synergistically merges the loop-slimming and high-polarization advantages characteristic of Al-centered modification routes with the leakage-suppression and high-field robustness typically achieved through Ta-centered strategies, while simultaneously employing multi-cation substitution to naturally drive microstructural refinement through solute drag mechanisms without requiring additional processing steps.

Structural characterization confirmed the formation of a homogeneous perovskite solid solution with progressive lattice contraction and enhanced microstrain, reflecting the successful incorporation of multi-scale ionic disorder at both A- and B-sites. This compositional disorder disrupts long-range ferroelectric ordering, promoting the formation of dynamic polar nanoregions that yield slim P-E hysteresis loops with minimized remnant polarization and reduced energy losses. The synergistic substitution strategy also suppresses oxygen vacancy formation through internal charge compensation between donor (Nb^5+^, Ta^5+^) and acceptor (Al^3+^) dopants while refining grain size to enhance breakdown strength via increased grain boundary scattering of charge carriers. These structural and electrical modifications work in concert to maximize the polarization difference while elevating the breakdown field, establishing optimal conditions for high-efficiency energy storage.

The optimized composition at x = 0.10 achieved a recoverable energy density of 3.357 J cm^−3^ with 90.5% efficiency at a moderate and practically accessible breakdown field of 240 kV cm^−1^, representing a 243% increase in *W_rec_* and 145% improvement in *η* relative to the undoped sample. Critically, these metrics were obtained at electric fields substantially lower than the 600–800 kV cm^−1^ typically required in reported high-performance systems, demonstrating a key practical advantage stemming directly from the synergistic A/B-site modification approach. The material exhibited robust operational stability with minimal performance variation across frequency (0.1–200 Hz), temperature (25–175 °C), and cycling (10^6^ cycles) tests, attributed to the thermal persistence of polar nanoregions that maintain diffuse dielectric response without macroscopic phase transitions. Charge–discharge evaluations further validated practical viability, delivering 0.826 J cm^−3^ discharged energy density at 90% efficiency within approximately 26 nanoseconds alongside charge and power densities of 582 A cm^−2^ and 46.6 MW cm^−3^, respectively.

These results establish that synergistic A/B-site co-modification, combining tolerance-factor engineering, compositional disorder induction, and internal defect compensation within a single system, provides a superior design framework for lead-free dielectric capacitors. The demonstrated balance of high energy storage density, exceptional efficiency under moderate operating fields, and robust stability positions this material platform as a promising candidate for practical pulsed power applications in electric vehicles, aerospace systems, and renewable energy storage technologies.

## Figures and Tables

**Table 1:** The effect of composition (x) on the bulk density, average grain size, and breakdown strength.

Composition (x)	Bulk Density (g cm^−3^)	Average Grain Size (μm)	Breakdown Strength (kV cm^−1)^
0	5.68	1.89	170
0.05	5.74	1.09	210
0.10	5.81	1.03	240
0.15	5.86	0.91	270

**Table 2:** Summary of energy storage performance parameters for (1 − x)BNTNb-xSBTA ceramics at breakdown electric field.

Composition (x)	*E_b_* (kv cm^−1^)	*W_rec_* (J cm^−3^)	*W_tot_* (J cm^−3^)	*η* (%)
0	170	0.976	2.647	36.9
0.05	210	2.463	4.564	53.9
0.10	240	3.357	3.711	90.5
0.15	270	2.372	2.759	85.9

## Data Availability

The raw data supporting the conclusions of this article will be made available by the authors on request.

## Supplementary Materials

The following supporting information can be downloaded at: https://www.mdpi.com/article/10.3390/ma18235259/s1, Figure S1. Enlarged SEM morphology and EDS mapping images of (1 − x)BNTNb-xSBTA (x = 0.1) ceramics. Figure S2. Leakage current density as a function of electric field for (1 − x)BNTNb-xSBTA ceramics with different compositions (x = 0, 0.05, 0.1, 0.15) measured at room temperature.

## References

[1] Chu S., Majumdar A. (2012). Opportunities and challenges for a sustainable energy future. Nature.

[2] Wang G., Lu Z., Li Y., Li L., Ji H., Feteira A., Zhou D., Wang D., Zhang S., Reaney I.M. (2021). Electroceramics for High-Energy Density Capacitors: Current Status and Future Perspectives. Chem. Rev..

[3] Li D., Zeng X., Li Z., Shen Z.-Y., Hao H., Luo W., Wang X., Song F., Wang Z., Li Y. (2021). Progress and perspectives in dielectric energy storage ceramics. J. Adv. Ceram..

[4] Jayakrishnan A.R., Silva J.P.B., Kamakshi K., Dastan D., Annapureddy V., Pereira M., Sekhar K.C. (2023). Are lead-free relaxor ferroelectric materials the most promising candidates for energy storage capacitors?. Prog. Mater. Sci..

[5] Zhang H., Wei T., Zhang Q., Ma W., Fan P., Salamon D., Zhang S.-T., Nan B., Tan H., Ye Z.-G. (2020). A review on the development of lead-free ferroelectric energy-storage ceramics and multilayer capacitors. J. Mater. Chem. C.

[6] Zhao P., Cai Z., Wu L., Zhu C., Li L., Wang X. (2021). Perspectives and challenges for lead-free energy-storage multilayer ceramic capacitors. J. Adv. Ceram..

[7] Zhou X., Xue G., Luo H., Bowen C.R., Zhang D. (2021). Phase structure and properties of sodium bismuth titanate lead-free piezoelectric ceramics. Prog. Mater. Sci..

[8] Pattipaka S., Lim Y., Son Y.H., Bae Y.M., Peddigari M., Hwang G.-T. (2024). Ceramic-Based Dielectric Materials for Energy Storage Capacitor Applications. Materials.

[9] Li D., Shen Z.-Y., Li Z., Luo W., Wang X., Wang Z., Song F., Li Y. (2020). P-E hysteresis loop going slim in Ba_0.3_Sr_0.7_TiO_3_-modified Bi_0.5_Na_0.5_TiO_3_ ceramics for energy storage applications. J. Adv. Ceram..

[10] Yan F., Huang K., Jiang T., Zhou X., Shi Y., Ge G., Shen B., Zhai J. (2020). Significantly enhanced energy storage density and efficiency of BNT-based perovskite ceramics via A-site defect engineering. Energy Storage Mater..

[11] Deng T., Liu Z., Hu T., Dai K., Hu Z., Wang G. (2023). Excellent energy-storage performance in Bi_0.5_Na_0.5_TiO_3_-based lead-free composite ceramics via introducing pyrochlore phase Sm_2_Ti_2_O_7_. Chem. Eng. J..

[12] Zhang A., Jing R., Zhuang M., Hou H., Zhang L., Zhang J., Lu X., Yan Y., Du H., Jin L. (2021). Nonstoichiometric effect of A-site complex ions on structural, dielectric, ferroelectric, and electrostrain properties of bismuth sodium titanate ceramics. Ceram. Int..

[13] Han F., Deng J., Liu X., Yan T., Ren S., Ma X., Liu S., Peng B., Liu L. (2017). High-temperature dielectric and relaxation behavior of Yb-doped Bi_0.5_Na_0.5_TiO_3_ ceramics. Ceram. Int..

[14] Zhou C.R., Liu X.Y. (2008). Effect of B-site substitution by (Ni_1/3_Nb_2/3_)^4+^ for Ti^4+^ on microstructure and piezoelectric properties in (Bi_1/2_Na_1/2_)TiO_3_ piezoelectric ceramics. J. Alloys Compd..

[15] Qiao Y., Li W., Zhang Y., Jing L., Yang X., Gao C., Cao W., Fei W. (2020). Ba and Mg co-doping to suppress high-temperature dielectric loss in lead-free Na_0.5_Bi_0.5_TiO_3_-based systems. J. Eur. Ceram. Soc..

[16] Yan F., Zhou X., He X., Bai H., Wu S., Shen B., Zhai J. (2020). Superior energy storage properties and excellent stability achieved in environment-friendly ferroelectrics via composition design strategy. Nano Energy.

[17] Fan X., Wang J., Yuan H., Zheng Z., Zhang J., Zhu K. (2023). Multi-scale synergic optimization strategy for dielectric energy storage ceramics. J. Adv. Ceram..

[18] Ma C., Tan X. (2011). In situ Transmission Electron Microscopy Study on the Phase Transitionsin Lead-Free (1 − x)(Bi_1/2_Na_1/2_)TiO_3_ − xBaTiO_3_ Ceramics. J. Am. Ceram. Soc..

[19] Kang R., Wang Z., Yang W., Zhu X., He L., Gao Y., Zhao J., Shi P., Zhao Y., Mao P. (2022). Enhanced energy storage performance in Sr_0.7_La_0.2_Zr_0.15_Ti_0.85_O_3_-modified Bi_0.5_Na_0.5_TiO_3_ ceramics via constructing local phase coexistence. Chem. Eng. J..

[20] Li Z., Li D.-X., Shen Z.-Y., Zeng X., Song F., Luo W., Wang X., Wang Z., Li Y. (2022). Remarkably enhanced dielectric stability and energy storage properties in BNT—BST relaxor ceramics by A-site defect engineering for pulsed power applications. J. Adv. Ceram..

[21] Yang F., Li Q., Zhang A., Jia Y., Sun Y., Wang W., Fan H. (2022). High energy storage density and temperature stable dielectric properties of (1 − x)Bi_0.5_(Na_0.82_K_0.18_)_0.5_Ti_0.99_(Y_0.5_Nb_0.5_)_0.01_O_3_–xNaNbO3 ceramics. J. Alloys Compd..

[22] Wang F., Xu M., Tang Y., Wang T., Shi W., Leung C.M. (2012). Large Strain Response in the Ternary Bi_0.5_Na_0.5_TiO_3_–BaTiO_3_–SrTiO_3_ Solid Solutions. J. Am. Ceram. Soc..

[23] Yan Y., Geng L.D., Zhu L.-F., Leng H., Li X., Liu H., Lin D., Wang K., Wang Y.U., Priya S. (2022). Ultrahigh Piezoelectric Performance through Synergistic Compositional and Microstructural Engineering. Adv. Sci..

[24] Xi J., Lin L., Bai W., Wu S., Zheng P., Li P., Zhai J. (2024). Compromise boosted high capacitive energy storage in lead-free (Bi_0.5_Na_0.5_)TiO_3_-based relaxor ferroelectrics by phase structure modulation and defect engineering. Chem. Eng. J..

[25] Xu K., Yang L., Wang J., Huang H. (2025). Design of high energy storage ferroelectric materials by phase-field simulations. J. Mater. Inform..

[26] Dai S., Li M., Wu X., Wu Y., Li X., Hao Y., Luo B. (2024). Combinatorial optimization of perovskite-based ferroelectric ceramics for energy storage applications. J. Adv. Ceram..

[27] Zhu W., Shen Z.-Y., Deng W., Li K., Luo W., Song F., Zeng X., Wang Z., Li Y. (2024). A review: (Bi,Na)TiO_3_ (BNT)-based energy storage ceramics. J. Mater..

[28] Feng M., Feng Y., Zhang T., Li J., Chen Q., Chi Q., Lei Q. (2021). Recent Advances in Multilayer-Structure Dielectrics for Energy Storage Application. Adv. Sci..

[29] Zubairi H., Lu Z., Zhu Y., Reaney I.M., Wang G. (2024). Current development, optimisation strategies and future perspectives for lead-free dielectric ceramics in high field and high energy density capacitors. Chem. Soc. Rev..

[30] Yan F., Qian J., Wang S., Zhai J. (2024). Progress and outlook on lead-free ceramics for energy storage applications. Nano Energy.

[31] Veerapandiyan V., Benes F., Gindel T., Deluca M. (2020). Strategies to Improve the Energy Storage Properties of Perovskite Lead-Free Relaxor Ferroelectrics: A Review. Materials.

[32] Luo C., Feng Q., Luo N., Yuan C., Zhou C., Wei Y., Fujita T., Xu J., Chen G. (2021). Effect of Ca^2+^/Hf^4+^ modification at A/B sites on energy-storage density of Bi_0.47_Na_0.47_Ba_0.06_TiO_3_ ceramics. Chem. Eng. J..

[33] Zhang X., Yang F., Miao W., Su Z., Zhao J., Tang L., Shen Y., Hu D., Chen Y., Li P. (2021). Effective improved energy storage performances of Na_0.5_Bi_0.5_TiO_3_-based relaxor ferroelectrics ceramics by A/B-sites co-doping. J. Alloys Compd..

[34] Xiong W., Zhang H., Cao S., Gao F., Svec P., Dusza J., Reece M.J., Yan H. (2021). Low-loss high entropy relaxor-like ferroelectrics with A-site disorder. J. Eur. Ceram. Soc..

[35] Li M., Li L., Zang J., Sinclair D.C. (2015). Donor-doping and reduced leakage current in Nb-doped Na_0.5_Bi_0.5_TiO_3_. Appl. Phys. Lett..

[36] Cen Z., Zhou C., Cheng J., Zhou X., Li W., Yan C., Feng S., Liu Y., Lao D. (2013). Effect of Zr^4+^ substitution on thermal stability and electrical properties of high temperature BiFe_0.99_Al_0.01_O_3_–BaTi_1−x_Zr_x_O_3_ ceramics. J. Alloys Compd..

[37] Wang J., Chen X.-M., Zhao X.-M., Liang X.-X., Zhou J.-P., Liu P. (2015). Effects of BiAlO_3_-doping on dielectric and ferroelectric properties of 0.93Na_0.5_Bi_0.5_TiO_3_–0.07BaTiO_3_ lead-free ceramics. Mater. Res. Bull..

[38] Yan F., Yang H., Lin Y., Wang T. (2017). Dielectric and Ferroelectric Properties of SrTiO_3_–Bi_0.5_Na_0.5_TiO_3_–BaAl_0.5_Nb_0.5_O_3_ Lead-Free Ceramics for High-Energy-Storage Applications. Inorg. Chem..

[39] Jin C.C., Wang F.F., Wei L.L., Tang J., Li Y., Yao Q.R., Tian C.Y., Shi W.Z. (2014). Influence of B-site complex-ion substitution on the structure and electrical properties in Bi_0.5_Na_0.5_TiO_3_-based lead-free solid solutions. J. Alloys Compd..

[40] Zhao Y., Xu J., Zhou C., Yuan C., Li Q., Chen G., Wang H., Yang L. (2016). High energy storage properties and dielectric behavior of (Bi_0.5_Na_0.5_)_0.94_Ba_0.06_Ti_1−x_(Al_0.5_Nb_0.5_)_x_O_3_ lead-free ferroelectric ceramics. Ceram. Int..

[41] Groszewicz P.B., Koch L., Steiner S., Ayrikyan A., Webber K.G., Frömling T., Albe K., Buntkowsky G. (2020). The fate of aluminium in (Na,Bi)TiO_3_-based ionic conductors. J. Mater. Chem. A.

[42] Jiang Z., Yang H., Cao L., Yang Z., Yuan Y., Li E. (2021). Enhanced breakdown strength and energy storage density of lead-free Bi_0.5_Na_0.5_TiO_3_-based ceramic by reducing the oxygen vacancy concentration. Chem. Eng. J..

[43] Qiao X., Liao A., Chen B., Zhang H., Zhang L., Kong T., Chao X., Yang Z. (2023). Enhanced energy storage properties of BNT-based ceramics via composition and multiscale structural engineering. Solid State Sci..

[44] Chen H., Shi J., Chen X., Sun C., Pang F., Dong X., Zhang H., Zhou H. (2021). Excellent energy storage properties and stability of NaNbO_3_–Bi(Mg_0.5_Ta_0.5_)O_3_ ceramics by introducing (Bi_0.5_Na_0.5_)_0.7_Sr_0.3_TiO_3_. J. Mater. Chem. A.

[45] Petnoi N., Bomlai P., Jiansirisomboon S., Watcharapasorn A. (2013). Effects of Nb-doping on the micro-structure and dielectric properties of (Bi_0.5_Na_0.5_)TiO_3_ ceramics. Ceram. Int..

[46] Zhang Z., Li Q., Zheng S., Chen X., Yang N., Yang Z., Shang K., Fan H., Wang W. (2025). Enhancement of energy storage performance in BNT-based energy ceramics via polar nanoregions induced by doping (Al_0.5_Ta_0.5_)^4+^ composite particles. Ceram. Int..

[47] Hiruma Y., Nagata H., Takenaka T. (2009). Thermal depoling process and piezoelectric properties of bismuth sodium titanate ceramics. J. Appl. Phys..

[48] Bhattacharyya R., Das S., Das A., Omar S. (2021). Effect of sintering temperature on the microstructure and conductivity of Na_0.54_Bi_0.46_Ti_0.99_Mg_0.01_O_3−δ_. Solid State Ion..

[49] Xia Y., Hao H., Tao C., Cao M., Yao Z., Liu H. (2025). Enhanced Energy Storage Performance in Mn-Doped SrBi_5_Ti_4_FeO_18_ Thin Films via Defect Engineering. ACS Appl. Mater. Interfaces.

[50] Cai Z., Wang X., Hong W., Luo B., Zhao Q., Li L. (2018). Grain-size–dependent dielectric properties in nanograin ferroelectrics. J. Am. Ceram. Soc..

[51] Yang Z., Du H., Jin L., Hu Q., Qu S., Yang Z., Yu Y., Wei X., Xu Z. (2019). A new family of sodium niobate-based dielectrics for electrical energy storage applications. J. Eur. Ceram. Soc..

[52] Cao W., Wu Y., Yang X., Guan D., Huang X., Li F., Guo Y., Wang C., Ge B., Hou X. (2025). Breaking polarization-breakdown strength paradox for ultrahigh energy storage density in NBT-based ceramics. Nat. Commun..

[53] Antillon E., Woodward C., Rao S.I., Akdim B. (2021). Chemical short range order strengthening in BCC complex concentrated alloys. Acta Mater..

[54] Wei T., Zhang Y., Kong L., Kim Y.J., Xu A., Karatchevtseva I., Scales N., Gregg D.J. (2019). Hot isostatically pressed Y_2_Ti_2_O_7_ and Gd_2_Ti_2_O_7_ pyrochlore glass-ceramics as potential waste forms for actinide immobilization. J. Eur. Ceram. Soc..

[55] Wohninsland A., Fetzer A.-K., Riaz A., Kleebe H.-J., Rödel J., Kodumudi Venkataraman L. (2021). Correlation between enhanced lattice distortion and volume fraction of polar nanoregions in quenched Na_1/2_Bi_1/2_TiO_3_–BaTiO_3_ ceramics. Appl. Phys. Lett..

[56] Manley M.E., Lynn J.W., Abernathy D.L., Specht E.D., Delaire O., Bishop A.R., Sahul R., Budai J.D. (2014). Phonon localization drives polar nanoregions in a relaxor ferroelectric. Nat. Commun..

[57] Wei K., Duan J., Qi H., Ma L., Du Q., Yu H., Wang H., Shi X., Li G., Shuai Z. (2025). Collaborative design of polarization and antiferrodistortion configurations in high energy capacitive relaxor ferroelectrics. Nat. Commun..

[58] Yin J., Shi X., Tao H., Tan Z., Lv X., Ding X., Sun J., Zhang Y., Zhang X., Yao K. (2022). Deciphering the atomic-scale structural origin for large dynamic electromechanical response in lead-free Bi_0.5_Na_0.5_TiO_3_-based relaxor ferroelectrics. Nat. Commun..

[59] Mendez-González Y., Peláiz-Barranco A., Curcio A.L., Rodrigues A.D., Guerra J.D.S. (2019). Raman spectroscopy study of the La-modified (Bi_0.5_Na_0.5_)_0.92_Ba_0.08_TiO_3_ lead-free ceramic system. J. Raman Spectrosc..

[60] Chu B., Hao J., Li P., Li Y., Li W., Zheng L., Zeng H. (2022). High-Energy Storage Properties over a Broad Temperature Range in La-Modified BNT-Based Lead-Free Ceramics. ACS Appl. Mater. Interfaces.

[61] Jiao Y., Song S., Chen F., Zeng X., Wang X., Song C., Liu G., Yan Y. (2022). Energy storage performance of 0.55Bi_0.5_Na_0.5_TiO_3_-0.45SrTiO_3_ ceramics doped with lanthanide elements (Ln = La, Nd, Dy, Sm) using a viscous polymer processing route. Ceram. Int..

[62] Liu G., Dong J., Zhang L., Yan Y., Jing R., Jin L. (2020). Phase evolution in (1 − x)(Na_0.5_Bi_0.5_)TiO_3_-xSrTiO_3_ solid solutions: A study focusing on dielectric and ferroelectric characteristics. J. Mater..

[63] Xu Q., Liu H., Zhang L., Xie J., Hao H., Cao M., Yao Z., Lanagan M.T. (2016). Structure and electrical properties of lead-free Bi_0.5_Na_0.5_TiO3-based ceramics for energy-storage applications. RSC Adv..

[64] Yan F., Tu Z., Wang W., Zhu Z., Chen Y., Liao J., Zhang S., Liao M., Zhou Y. (2025). Improving the ferroelectric and dielectric properties of barium strontium titanate thin films via local chemical design. Inorg. Chem. Front..

[65] Li Z., Zhou W., Guo J., Lv X., Lin S., Li Y., Gu L., Yan F. (2025). Outstanding comprehensive energy storage performance in BNT-based lead-free ceramics via synergistic optimization strategy. Chem. Eng. J..

[66] Zhou X., Huang Z., Luo X., Zhang J., Li Y., Zhou X., Li M., Zhang D. (2025). Bi_0.5_Na_0.5_TiO_3_-Based Relaxor Ferroelectrics with Highly Polarizable Concentrated Nanodomains for High Capacitive Energy Storage under Moderate Electric Fields. ACS Appl. Mater. Interfaces.

[67] Chen F., Yang L., Feng H., Li Q., Zeng X., Yu K., Song C., Yan Y., Jin L., Zhang D. (2024). An ultrahigh energy storage efficiency and recoverable density in Bi_0.5_Na_0.5_TiO_3_ with the modification of Sr_0.85_La_0.1_TiO_3_ via viscous polymer process. J. Mater..

[68] Shi W., Zhang L., Jing R., Hu Q., Zeng X., Alikin D.O., Shur V.Y., Wei X., Gao J., Liu G. (2022). Relaxor antiferroelectric-like characteristic boosting enhanced energy storage performance in eco-friendly (Bi_0.5_Na_0.5_)TiO_3_-based ceramics. J. Eur. Ceram. Soc..

