# Supplementary material for: High-Efficiency Lead-Free BNT-Based Relaxor Ferroelectrics via Synergistic A/B-Site Substitution for Enhanced Energy Storage and Stability
Source: Materials (Basel). 2025 Nov 21;18(23):5259. doi: 10.3390/ma18235259 (PMC12693421; doi:10.3390/ma18235259)
Supplement: Supplementary file 1 [file materials-18-05259-s001.zip › materials-3906440-supplementary.pdf]

High-Efficiency Lead-Free BNT-Based Relaxor Ferroelectrics via Synergistic A/B-Site Substitution for Enhanced Energy Storage and Stability

Wenjie Zhou [1], Tao Du [1,*] and Changbai Long [2,*]

1 School of Advanced Materials and Nanotechnology, Xidian University, Xi'an 710126, China; 23141110645@stu.xidian.edu.cn

2 State Key Laboratory for Mechanical Behavior of Materials, Xi'an Jiaotong University, Xi'an 710049, China

* Correspondence: taodu@xidian.edu.cn (T.D.); longchangbai@xjtu.edu.cn (C.L.)

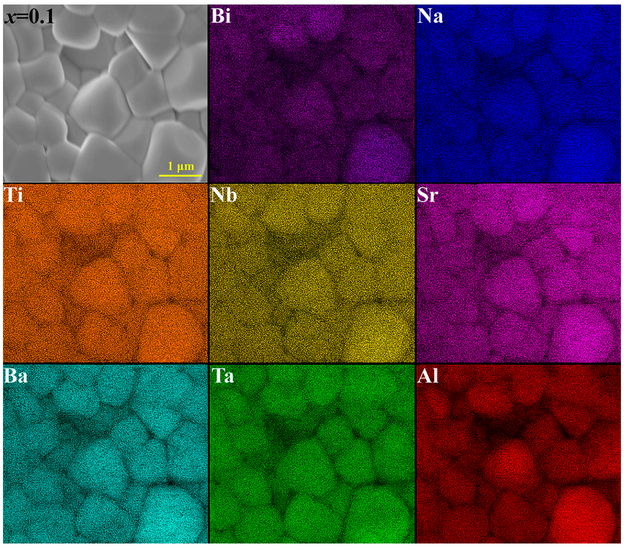

Figure S1. Enlarged SEM morphology and EDS mapping images of (1-x)BNTNb-xSBTA (x=0.1) ceramics.

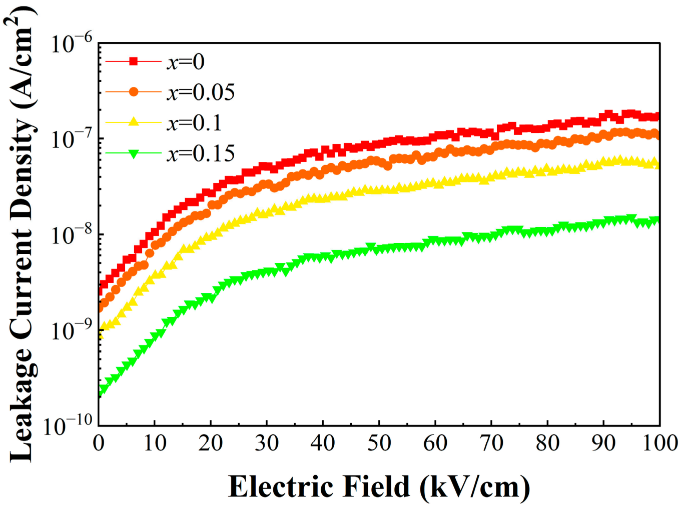

Figure S2. Leakage current density as a function of electric field for (1-x)BNTNb-xSBTA ceramics with different compositions (x = 0, 0.05, 0.1, 0.15) measured at room temperature.
